# Pakistan’s 2025 HPV Vaccine Phase I Rollout: Community Response, Implementation Challenges & Way Forward

**DOI:** 10.3390/vaccines14060537

**Published:** 2026-06-17

**Authors:** Wei Xia, Soofia Yunus, Atta Ur Rehman, Shah Nawaz Jiskani, Muhammad Imran Qureshi, Shawana Farooq, Inam Bhatti, Sunday Audu, Syed Natiq Abbas Kazmi, Rozina Khalid

**Affiliations:** 1 WHO Country Office, Prime Minister’s National Health Complex, Park Road, Chak Shahzad, Islamabad 44000, Pakistan; attaurrehman1987@yahoo.com (A.U.R.); jiskanis@who.int (S.N.J.); audusu@who.int (S.A.);; 2 Federal Directorate of Immunization (FDI), Prime Minister’s National Health Complex, Park Road, Chak Shahzad, Islamabad 44000, Pakistan

**Keywords:** HPV vaccine, national coverage, community engagement, implementation challenges, routine immunization

## Abstract

Background: The International Agency for Research on Cancer estimated around 3197 annual deaths along with 5008 newly diagnosed cases of cervical cancer in Pakistan. Worldwide, introduced in 164 WHO member states, the HPV vaccine provides over ninety percent (90%) protection from human papillomavirus (16 & 18 types) infections. This article intended to document the vaccine (HPV) introduction in a low-middle-income country through the lens of EPI preparedness, vaccination coverage achieved, community acceptance, and implementation challenges during Phase I. Methodology: The research applied a qualitative and quantitative mix method to review the intricate procedure of new vaccine rollout within the national context. A qualitative participant observation approach assessed the planning, approval, and implementation phases of the HPV vaccine. Quantitative data statistics were evaluated for national & regional vaccination coverages, rapid convenience assessment findings, and adverse events reports. Results: The overall reported administrative HPV campaign coverage was 75%, with the maximum regional coverage of 81% by the Punjab, followed by 66% of the Sindh, 43% by the Azad Jammu & Kashmir, and 38% by the Islamabad. Rapid Convenience Assessment findings highlighted the main reasons for refusal (71%), with unavailable girls during the campaign (22%) for non-HPV vaccination. Community acceptance varied across the regions, with notable challenges in implementation being observed. Discussion & Way Forward: Initial phase campaign coverage (70.6%) was greater than the worldwide reported first dose mean coverage (61.6%) for the same multi-age cohort, indicative of an encouraging start in resource limited setting. Documented coverage was below the high-performing countries but comparable to multiple low and middle-income countries. Federal Directorate of Immunization, in collaboration with provincial EPI stakeholders, should prioritize including the newly introduced HPV vaccine in the routine immunization schedule of the Phase I regions and should also implement the lessons learned in the subsequent rollout phases in 2026 in Khyber Pakhtunkhwa and 2027 in Balochistan & Gilgit Baltistan. Expanding fixed EPI sites for HPV vaccination, promoting school-centered vaccination, rationalizing outreach in marginalized areas, sustaining the cold chain system, implementing a culturally acceptable communication plan, and resolving internet connectivity challenges are the key strategies to address implementation challenges.

## 1. Introduction

Cancer of the cervix is a major global public health challenge, being the fourth most prevalent cause of cancer-related deaths among women [1,2]. In 2022 alone, an estimated 660,000 new cases and approximately 350,000 deaths were reported globally, with most of the cases from low-middle-income countries (LMICs) [3]. It is the sixth most prevalent cancer among females in the EMR (Eastern Mediterranean Region), with an estimated 89,800 newly diagnosed cases, and 47,500 reported deaths in the year 2020 [4]. The International Agency for Research on Cancer reported around 3197 annual deaths along with 5008 newly estimated cases of cervical cancer in Pakistan in 2023 [5]. The projected mortality rates of 60% to 85% remain considerably high, exceeding the global average of 45% [6,7].

Persistent HPV infections are responsible for over 99% of cervical cancer cases, with HPV subtypes (16 & 18) accounting for 88.1% of invasive cervical cancers in Pakistan [8,9]. Approximately 7.6% of Pakistani women were infected with HPV oncogenic types (16 & 18). Human papillomavirus is not only the causative agent of the most prevalent cervical cancer but also causes other cancers of the female reproductive system (vulva, vagina), male reproductive system (penis), oropharynx, and anus, with the majority of cancers reported (>90%) in females [7,10].

The World Health Assembly endorsed the WHO global strategy for cervical cancer prevention and elimination as a public health problem [1]. The WHO Global Strategy outlines the following 90-70-90 targets:

- 90% of girls are fully vaccinated against HPV by the age of 15 years
- 70% of women screened with a high-performance test between the ages of 35 & 45 years
- 90% of women diagnosed with cervical disease are receiving appropriate treatment

The Eastern Mediterranean region strategy promotes this aim by targeting a 30% decrease in cervical cancer fatalities by 2030 in the region using a triple-intervention approach, which is aligned with Sustainable Development Goal 3.4 [11]. The recommended prophylactic approach remains HPV vaccination, which provides over ninety percent (90%) protection from human papillomavirus (16 & 18 types) infections [12,13]. The Strategic Advisory Group of Experts on Immunization endorses routine vaccination of girls aged 9–14 years, ideally before exposure to the virus. Secondary targets may include boys and older age groups in specific contexts [14].

As of 2025, more than 160 out of 194 WHO member states have introduced HPV vaccines fully or partly into their national immunization schedules [15,16]. The Expanded Programme on Immunization (EPI) of Pakistan has not yet included the HPV vaccine into its routine national immunization schedule. The Federal Directorate of Immunization has taken practical measures to address this gap by aligning national immunization goals with the regional and global WHO guidelines through the technical assistance of coalition partners. Phase I of Pakistan’s national HPV vaccination campaign was launched in September 2025, which targeted school and out-of-school girls 9 to 14 years of age. Strong political commitment at national and provincial levels, extensive planning including technical working group meetings, national burden evidence critical analysis, engagement of global, regional, and local technical experts, NITAG and NICC approvals, and EPI program readiness reviews preceded the launch. This review observes the Phase I HPV vaccine introduction, aiming to evaluate the EPI program preparedness, coverage achieved, community acceptance, and implementation challenges. This article is intended to document the new vaccine introduction in a low-middle-income country and to utilize the key lessons learned for the implementation in the subsequent rollout phases in 2026 & 2027.

## 2. Methodology

This research applied a qualitative and quantitative mix method to review the intricate procedure of new vaccine rollout within the national context. A qualitative participant observation approach assessed the planning, approval, and implementation phases of the HPV vaccine across the Federal Directorate of Immunization hierarchy. WHO EPI team members observed the rollout purposely. The non-random purposive sampling technique was adopted to visit EPI sites, participate in technical working group meetings in FDI, NITAG, and NICC, and observe respondents in the community. Observational notes were drafted from formal meetings, field visits, trainings, minutes of meeting and informal deliberations with FDI staff, health care professionals, education department representatives, parents, adolescent girls, and teachers. Moreover, the media was screened to evaluate the infodemic, community narratives, and supportive environment during HPV vaccine introduction.

Quantitative data statistics were evaluated for the nationwide burden of cervical cancer, national & regional vaccination coverages, rapid convenience assessment findings, and Adverse Events Following Immunization (AEFI) reports. Data was presented in descriptive form, representing frequencies and percentages in tabular form. Numerical statistics & field observations portrayed the real snapshot to triangulate information to identify challenges with a deep insight into local social dynamics influencing acceptance and vaccine uptake. Ethical protocols were meticulously adhered to during this investigation. The Health Services Academy’s ethical review board approved the cervical cancer burden of disease national assessment. Respondents’ anonymity and information confidentiality were maintained during the documentation of the thematic analysis into narrative form. Research observers continued a reflexive approach, recognizing the impact of their observer and participant roles, which improved the transparency of the investigation findings and reduced biases.

## 3. Results

This section presents the main outcomes derived from the new vaccine (HPV) introduction in the country, reviewing the national introduction timeline (2023–2027), evaluating the national cervical cancer burden in the country, and assessing the EPI program readiness in selected provinces & federating areas. Coverage statistics from the national HPV vaccination campaign executed from 15–27 September 2025 were presented to view the coverage in various regions. The outputs of Rapid Convenience Assessments (RCA) with documented reports of Adverse Events Following Immunization (AEFI) were compiled to evaluate vaccination safety with coverage validity. The finding section lastly summarizes the implementation challenges during the campaign, general population response, analyzing HPV vaccine acceptance & refusals in the community with lessons learned that will guide subsequent introduction phases in Khyber Pakhtunkhwa, Gilgit Baltistan, and Balochistan.

### 3.1. Timeline of HPV Vaccine Introduction in Pakistan (2023–2027)

**Figure 1:**
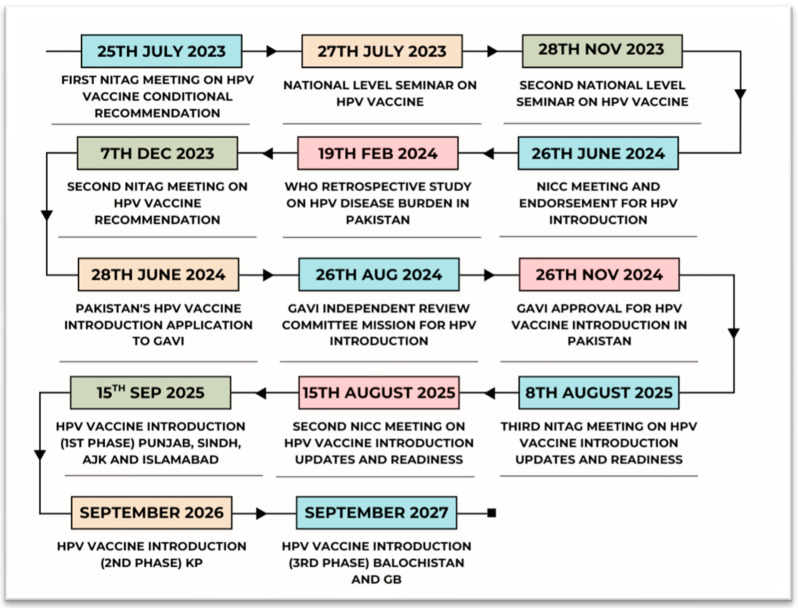
HPV Vaccine National Introduction Timeline (2023–2025).

The HPV vaccine was introduced in Pakistan using a planned and stepwise approach that included various levels of stakeholder involvement, technical evaluations, global collaboration, and EPI Program readiness assessments across regions. NITAG (National Immunization Technical Advisory Group) made a conditional recommendation at its preliminary meeting (25 July 2023) to start the initial planning for the country’s HPV vaccine introduction. The first step in this process recommended was to conduct a retrospective study of the country’s cervical cancer burden of disease over the previous three years (2021 to 2023). The Provincial Health Ministers (27 July 2023) and the Federal Health Ministers (28 November 2023) presence at the advocacy sessions reaffirmed the political assurance for the HPV vaccine introduction. NITAG follow-up consultation (7 December 2023) assessed the preconditioning proposal in relation to the available literature. The WHO CO informed the pertinent stakeholders in February 2024 of the findings of the retrospective burden survey. The National Interagency Coordination Committee approved the formal introduction of the HPV vaccine in the country (26 June 2024). The Federal Directorate of Immunization successively submitted a proposal for the new (HPV) vaccine introduction in the country (28 June 2024). Gavi’s Independent Review Committee delegation visited (on 26 August 2024) to check preparedness, and on 26 November 2024, final approval was given for the new vaccine rollout. National readiness was evaluated at the third NITAG meet on 8 August 2025, and programmatic as well as operational preparedness were examined at the second NICC meet on 15 August 2025. The nation-wide HPV vaccination campaign’s first phase started on 15 September 2025, in Punjab, Sindh, Islamabad, and Azad Jammu & Kashmir, and ended on 27 September 2025. The second phase of the HPV vaccination rollout is planned for September 2026 in Khyber Pakhtunkhwa (KP), and the last phase is scheduled for September 2027 in Gilgit-Baltistan (GB) and Balochistan, as presented in Figure 1.

### 3.2. Cervical Cancer Burden of Disease Assessment

#### 3.2.1. Cancer Registries Review of Literature

**Figure 2:**
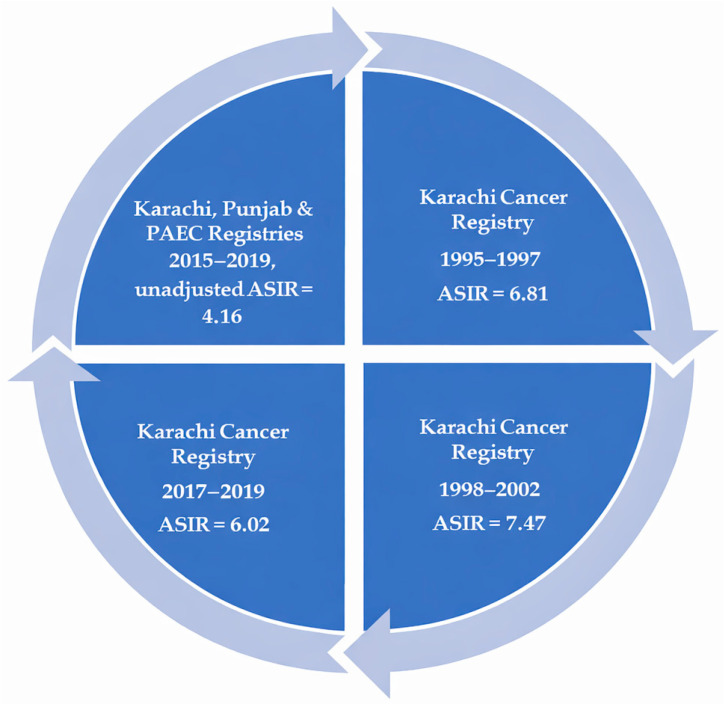
Age-Standardized Incidence Rates of cervical cancer in Pakistan.

A comprehensive literature review of the past three decades was conducted to estimate the age-standardized incidence rates (ASIR) of cervical cancer in Pakistan. Model assumptions were estimated based on data from thirteen studies. Karachi Cancer Registry reported the highest cervical cancer burden for all periods with ASIR of 6.81 (1995–1997), 7.47 (1998–2002), and 6.02 (2017–2019) per 100,000 women. Cancer registry data (2015 to 2019) from Punjab, Karachi, Pakistan Atomic Energy, and other healthcare facilities calculated ASIR of 4.16 per 100,000 women as shown in Figure 2. Model assumptions estimated ASIRs range from 5.2 to 8.4 per 100,000 women. The average new cases were calculated to be 6166 with a derived ASIR of 7.60. The estimated Age-standardized Incidence Rates (ASIR) of cervical cancer in Pakistan remain higher than the WHO target [7].

#### 3.2.2. Retrospective Data Analysis

The response rate was 32.72%, with data reported by 18 of 55 healthcare facilities contacted. There were 1580 reported cases of cervical cancer in the responding hospitals and cancer registry records from 2021 to 2023 in Pakistan, as shown in Table 1.

Only 18 healthcare facilities provided the data for analysis, so these survey results should be interpreted with caution as they present only limited data and do not portray the hidden burden of cervical cancer cases reported in other healthcare facilities. A noteworthy outcome from this survey was the lack of standardized, structured tools (Can Screen 5) across these institutions for reporting cervical cancer cases.

### 3.3. HPV Readiness Assessment in Selected Regions (May–September 2025)

**Figure 3:**
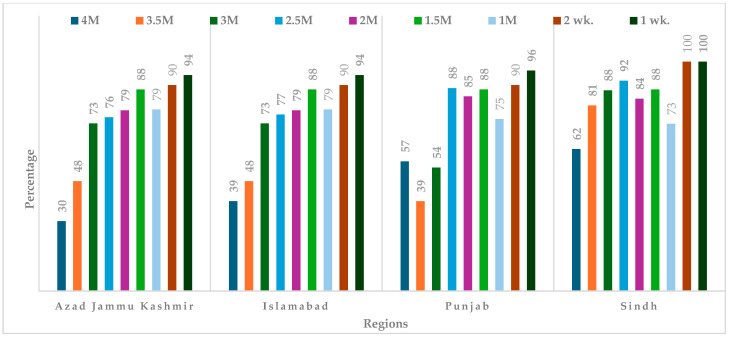
HPV Readiness Assessment in Selected Regions (May–September 2025).

EPI program readiness assessment covered several critical areas of microplanning, government & stakeholder commitment, logistics, community mobilization, monitoring, and evaluation to ensure effective implementation for new vaccine introduction (HPV). The progress was tracked at multiple checkpoints (May–September 2025) to monitor trends aimed at corrective measures, as deemed necessary, as shown in Figure 3.

The initial assessment findings highlight challenges at the province, district, and union council levels linked to micro plans, government ownership, community mobilization, and multisectoral collaboration. The final evaluation on 8 September 2025 showed 94% readiness in Azad Jammu & Kashmir, 96% readiness in Islamabad and Punjab, 100% readiness in Sindh, with an average of 94% readiness at the national level, highlighting notable progress in all indicators. Over 49,000 healthcare professionals received preliminary training, which heightened the pilot provinces’ preparedness prior to scaling up.

### 3.4. Coverages Achieved During HPV Campaign (15–27 September) & Catch-Up Days

The Federal Directorate of Immunization, in partnership with provincial health departments and with technical assistance from WHO and other partners, launched HPV campaign Phase I to prevent cervical cancer in Punjab, Sindh, AJK, and Islamabad from 15 to 27 September, targeting girls aged 9–14 years, both in and out of school.

The HPV vaccination campaign was extended by three days, from 29 September to 1 October 2025, and implemented across all provinces. In total, 9.7 million eligible girls (9 to 14 years) were vaccinated with cecolin single dose during the national twelve campaign days and three additional catch-up days. The maximum number of 6.87 million girls were vaccinated in Punjab, followed by 2.67 million girls in Sindh, 116.7 thousand in AJK, and almost 56 thousand in Islamabad.

The national HPV vaccination coverage was 75%, with the maximum regional coverage of 81% by the Punjab, followed by 66% of the Sindh, 43% by the Azad Jammu & Kashmir, and 38% by the Islamabad, as shown in Table 2.

Seventy-eight districts were categorized according to their vaccination coverage achieved during both the campaign and catch-up phases. In Punjab province, out of the 36 districts, 29 districts achieved 80% and higher coverage, while 7 districts reported coverage in the range of 70% to 79%. Sindh, with a total of 30 districts, 17 districts reached 80% and higher coverage, while 4 of the districts were in the range of 70% to 79%, 2 of the districts were in the range of 60% to 69%, whereas 7 of the districts had reported coverage below the 60%. In Azad Jammu & Kashmir, out of the 10 districts majority (9) were below 60%, and 1 district reported coverage between 60% to 69%. The federal capital, Islamabad, and both districts reported coverage below 60%. In total, 46 districts across the nation have coverage of 80% and more, 11 have coverage from 70% to 79%, 3 have coverage between 60% to 69%, and 18 have less than 60% overall coverage as presented in Table 3.

There were 4,948,317 missed girls in total, including 881,114 unavailable girls, 4,018,564 refusals, and 48,639 ill girls, of whom 1,146,908 (23.1%) were only vaccinated following a follow-up consultation, as shown in Table 4.

The vast majority (65.7%) of the estimated 9.7 million girls who received the HPV vaccination throughout the campaign, as well as catch-up days, received it via community-based vaccination practices, as shown in Table 5.

Rapid Convenience Assessment (RCA) is a structured tool used during the campaign to evaluate immunization coverage while identifying reasons behind unvaccinated and missed girls instantly after the HPV campaign. Only 54% (51,450) RCAs were conducted, with 37% (18,947) identified areas for mop-up activities as presented in Table 6 below.

From the sampled population, sources of information for the HPV vaccination campaign included health workers (31%), mosque miking (21%), mobile miking (18%), posters (9%), school children (8%), television (7%), newspapers (4%), and other sources (2%). Among unvaccinated girls in the sampled population, the main reasons for non-immunization were refusal (71%), unavailability of the girl at the time of vaccination (22%), illness (3%), other reasons (2%), lack of parental awareness (1%), and absence of HPV campaign information (0%), as shown in Table 7.

There were 190 adverse events following immunization reported overall during the campaign, with the most frequent being nausea and vomiting, followed by headaches, swelling, redness at the injection site, loss of consciousness, fever over 38 °C, diarrhea, injection site abscess, and rash. Table 8 presents the distribution and classification of these adverse events, providing an overview of their types and severity across all reporting areas.

### 3.5. Provincial Distribution of Burden of Disease vs. HPV Vaccination Coverage

**Figure 4:**
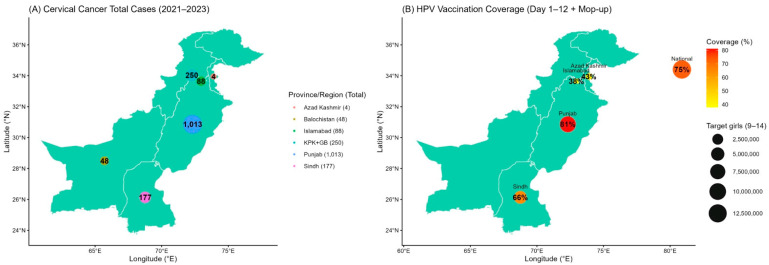
Comparison of cervical cancer burden (2021–2023) and HPV vaccination campaign coverage across provinces.

Figure 4 presents a comparative overview of the facility-reported cervical cancer burden during 2021–2023 (Figure 4A) and HPV vaccination campaign coverage (Figure 4B) across provinces and regions of Pakistan. When viewed together, the figure enables a comparative assessment of disease burden and vaccination performance, showing that provinces with a higher reported burden of cervical cancer also achieved relatively higher campaign coverage, although this pattern is not uniform across all regions. The figure underscores provincial heterogeneity in both reported disease burden and vaccination coverage, highlighting areas where intensified programmatic focus may be required.

### 3.6. Community Response

In Pakistan, there have been mixed community responses to the HPV vaccine, with noticeable disparities across provinces & federal administered areas. Although certain communities demonstrated encouraging attitudes with overwhelming acceptance, others raised concerns about social, cultural, faith-related, and false infodemic perspectives. Field monitors observed resistance and refusals in a number of districts, stemming from misunderstandings regarding fertility, concerns about the safety of the HPV vaccine, and a lack of knowledge about HPV and its association with cervical cancer. Districts & union councils that were well sensitized through social mobilizers, medical professionals, educational institutions, as well as neighborhood influencers, on the other hand, showed higher acceptance and engagement.

During the national rollout, considerable variation in HPV vaccine coverage was evident across provinces. Field observations & rapid convenience assessment findings also confirmed variation in community acceptance rates. Numerous refusal categories were identified predominantly in low coverage areas of Islamabad, Azad Jammu & Kashmir, Sindh, and Punjab. Thematic analysis of determinants affecting vaccine acceptance in the community identified themes about HPV vaccine safety perceptions, religious & cultural opinions, moral & communal concerns, mistrust & disinformation impact, and social taboos about STIs.

#### 3.6.1. HPV Vaccine Safety Perceptions

The most common reason for the HPV vaccine refusal observed was concerns regarding vaccine safety with possible adverse effects. Many parents were concerned that this HPV vaccine might induce infertility, affect their girls’ reproductive systems, or possibly be part of a hidden plan for reducing the population of the Muslim community. Several feared the vaccination would cause instant side effects such as fever, dizziness, seizures, and potentially death, with some describing the HPV vaccine as “a poison being administered to my daughter”.

#### 3.6.2. Religious & Cultural Opinions

Concerns related to religious beliefs also played a significant role in vaccine hesitancy. Many parents believed that God had predetermined each person’s health and illness, leading them to view vaccinations and other preventive measures as unnecessary. Many concerns were raised about the prohibited ingredients in the vaccine, including alcohol and pork products. Some were concerned that immunization might promote promiscuity, with a few parents claiming that the vaccine would cause their daughters to violate family norms by participating in immoral sexual activities. “She will be actively engaged in sexual activity after HPV vaccination”. A common assumption was that cervical cancer was not perceived as prevalent in their cultural context, with some believing that cancer only affects women in other countries, as their daughters were not at risk because they were religious and virgins.

#### 3.6.3. Moral & Communal Concerns

Communal taboos about sexuality have hindered acceptance, as treating/disclosing sexually transmitted infections (STIs) was considered deplorable, principally for teenage, unmarried girls. In certain rural families, immunizing females against STIs was considered disrespectful and an insult to their dignity, potentially harming the family’s image. Social expectations, “What people will think about us,” and criticism from local representatives, “No one will marry your daughter after getting this vaccination,” prevented certain parents from immunizing their daughters with the HPV vaccine. Gender prejudice caused numerous families to neglect their girls’ health, which rendered them more reluctant to get preventative treatment.

#### 3.6.4. Mistrust & Disinformation Impact

The lack of trust in the government, health system, and international organizations increased dissent. Several parents considered the vaccine in the context of a West conspiracy to damage Muslims, whereas some suspected that the authorities had hidden intentions, as well as were not operating in the best interests of their citizens. Several individuals voiced concern that their girls were acting as “guinea pigs” to test experimental vaccines, citing previous interactions with substandard medical care. Disinformation linked to HPV vaccines circulated swiftly via online databases, showbiz personalities, and influential persons, resulting in community doubts and refusals.

### 3.7. Implementation Challenges

Notable implementation challenges were identified during the national vaccine rollout about HPV vaccine coverage variation & uptake, managerial & departmental challenges, geographical disparities & operational barriers, socio-religious factors, awareness & communication gaps, and data monitoring.

Overall HPV campaign vaccine coverage (75%) was below the benchmark of 90% for the targeted multiage cohort (9 to 14 years). Out of the total 78 districts, less than 70% percentage was documented in 21 districts of Sindh, AJK, and Islamabad. Low immunization uptake, with significant statistics of girls missed throughout the campaign and catch-up days, was recorded in these districts, highlighting persistent challenges in implementation. Refusals, non-availability, and sickness contributed to about 4.9 million missed girls across the country, reflecting gaps in social mobilization, administrative follow-up, and intersectoral teamwork. The preliminary phase experienced managerial & departmental challenges. Weak interdepartmental collaboration, political association, and security challenges interrupted consistent vaccine delivery and precipitated community refusals. Minimal engagement with academic institutions, religious scholars, government officials, media representatives, and social influencers compromised campaign acceptability through stakeholders’ ownership. Communication failures, especially the Punjab Provincial educational department sending out parental consent form a single day prior to the initiative, resulted in missed opportunities and delayed sessions affecting vaccine uptake. Scheduled vaccination sessions have been canceled in urban slums, especially in private institutions, due to limited EPI staff access.

Provincial disparities with operational limitations in overdue consignments and temperature fluctuations were evident in flood-affected districts, mountainous areas, and remote regions EPI program’s predominant reliance on school-centered vaccination approach and outreach activities, alongside suboptimal utilization of fixed locations, missed migrant residents, and unenrolled school girls. Additionally, as the vaccine is to be included in the routine immunization schedule, it will become difficult to convince people to seek services from fixed centers. Lady Health Workers were not actively engaged in the campaign in the majority of the districts that affect vaccine uptake, as they are considered trustworthy in the community.

Socio-religious challenges affected the demand generation in the society. Parental refusals with HPV vaccine hesitancy were observed due to concerns about vaccine safety, fertility, potential non-halal or harmful substances in the Cecolin vaccine, and religion not allowing vaccination as acceptable. Delayed social mobilization, education department coordination, parental engagement, and crisis communication plan resulted in maximum refusal rates in certain districts.

Lack of awareness about the HPV vaccine and gaps in the communication strategy created doubt in parts of the community about the campaign. Rumors and misinformation influenced the educated and uneducated populations equally. In a number of schools, misinformation among teaching staff led to schools refusing to cooperate and minimal coverage among girls attending these institutions. Additionally, a significant number of households were unaware of the campaign until vaccination teams approached for outreach activities, leading to misunderstandings and thus revealing gaps in the campaign communication plan. The population’s awareness became lower when false information was permitted to propagate widely due to the poor visibility of ads in traditional as well as online platforms.

Challenges in data monitoring involved limited real-time insight into vaccine acceptance and refusal statistics, hindered by the low completion rate and incomplete reporting of Rapid Convenience Assessments (RCA) in some regions. Data submissions were also delayed due to internet connectivity issues, preventing underperforming regions from taking timely remedial actions. Last but not least, budgetary issues, such as the EPI initiatives’ dependence on funding from donors, might compromise routine vaccination, prospective scalability, operating continuity, and sustainability in subsequent phases.

## 4. Discussion

The HPV vaccine was first introduced in developed countries in 2006, expanded to 100 countries by 2019, and reached over 160 by 2025, with Pakistan joining the global community by launching its national introduction in September 2025. A literature search from 2024 revealed that many African countries took more than a decade to introduce the HPV vaccine nationally, despite its availability in the market since 2006, similar to the case of Pakistan [17]. The national rollout of the HPV vaccine served as a significant demonstration of adolescent immunization, as well as school-based vaccination, in the challenging setting of a developing country and marks a remarkable achievement in the country’s EPI history. Particularly in comparison to other countries where preliminary phases usually take three to five years before national implementation [17,18], Pakistan’s first Phase I (2023–2025) national launch timeline of two years and one month for the HPV vaccine introduction represents a notably rapid timeline in terms of planning, procurement, and readiness assessment for rollout. New vaccine (HPV) readiness evaluation before the campaign showed an average of 94% at the national level, 100% readiness in Sindh, 96% readiness in Islamabad and Punjab and 94% readiness in Azad Jammu & Kashmir, highlighting notable progress in all modules of planning, coordination & funding, advocacy social mobilization and communication, vaccine cold chain & logistics and monitoring & supervision. However, despite this EPI program preparedness, there were noteworthy geographical variations in the HPV vaccine coverage.

The HPV vaccine coverage was 75%, with the maximum regional coverage of 81% by the Punjab, and the minimum regional coverage of 38% by the Islamabad. National & regional coverages were less than the targeted standard of 90% coverage, reflecting implementation challenges in rollout. Initial phase national coverage (75%) was greater than the worldwide reported first dose mean coverage (61.6%) for the same multi-age cohort (9 to 14 years), indicative of an encouraging start in resource limited setting. Administrative coverage was below the high-performing countries but comparable to multiple low-middle-income countries. Massive variations were noted between countries in HPV vaccination acceptance, with the majority of high-income countries reporting higher HPV vaccine coverage rates, but countries like Japan and the Bahamas (<5%) also showed low coverage. Similarly, some of the developing countries reported better coverages (90 to 95%), like Tanzania, Bangladesh, and Bhutan, while other countries documented low coverages (<5%), such as Serbia, Morocco, and Qatar, demonstrating significant disparities in vaccination coverage [15,19]. These global comparisons reveal that particular variables ought to be improved for the HPV vaccine introduction in a country to be sustained. Adverse Events Following Immunization (AEFI) reported in the country were comparatively low during the campaign and catch-up days. Mild reactions were identified, validating the HPV Vaccine (Cecolin) safety profile and aligned with global documented statistics from WHO & other investigations reporting few adverse events [20,21]. HPV vaccine’s most frequent side effects reported were Nausea and Vomiting in Pakistan; however, in comparison to Bangladesh, for the same multi-age cohort, injection site pain was observed with the same brand [21].

Refusals with community hesitancy for their daughters to be vaccinated with the HPV vaccine continued to be a significant challenge throughout the rollout from mega cities like Karachi & Islamabad to rural zones of AJK & Punjab. Tanzania & Kenya also reported similar challenges of infertility, concerns about the safety of the HPV vaccine, and moral & communal concerns as observed in Pakistan [22,23]. Phase I experiences highlighted the significance of timely, continuous, and tailored communication tactics in countering disinformation during vaccine introduction. Infodemics with the new vaccine rollout were quite evident globally, with information reliance on reliable sources lessening hesitancy among the population; however, social media use only for vaccine statistics resulted in increased resistance [24]. School-centered vaccination strategies not only detect communication challenges in Pakistan, but other countries also reported similar patterns. Lessons from Bangladesh, Ethiopia, and Canada emphasized that improving HPV vaccine uptake in schools requires a strong public health communication approach and strengthened collaboration among the education and healthcare departments [25,26].

Digital monitoring & reporting challenges due to poor internet networks in AJK & remote areas were also evident in African countries (Ghana, Kenya). The operation of the EPI program may be affected by limited internet access, especially in rural and remote zones where delayed data uploading prevents prompt oversight and remedial action [27,28,29].

Public trust in the HPV vaccine (Cecolin) safety was increased by vigorous promotion and observable governance by national as well as provincial government officials, such as the nation’s health minister and other EPI officials, openly immunizing their own children during the second week of the campaign. Literature evidence from Cameroon also supports the notion that strong advocacy through trusted messengers helped increase community engagement & improved coverage. The majority population province of Punjab & certain districts of other regions, during the campaign last week & catch-up days, significantly improved their coverage [30].

## 5. Conclusions

In conclusion, Pakistan’s HPV vaccination introduction campaign, Phase I, demonstrated achievements in governance engagement, program readiness, and optimum HPV vaccine coverage at the national level despite continuous school engagement and community acceptance challenges. The HPV vaccine rollout in Pakistan highlights an important lesson: attaining high operational readiness alone, without the support of ongoing community trust and educational institutions, does not ensure high coverage in all areas. The program must give top priority to ongoing engagement with society & educational institutions, offline-capable monitoring of information tools, surveillance mechanisms, and focused outreach efforts headed by reliable community leaders in order to reach and sustain rates of coverage that exceed 90% in routine immunization and subsequent rollout phases in Khyber-Pakhtunkhwa, Balochistan, and Gilgit Baltistan.

## 6. Way Forward

Federal Directorate of Immunization, in collaboration with provincial EPI stakeholders, should prioritize including the newly introduced HPV vaccine in the routine immunization schedule of the Phase I piloted regions. They should also implement the lessons learned in the subsequent rollout phases in 2026 in Khyber Pakhtunkhwa and 2027 in Balochistan & Gilgit Baltistan. Community acceptance may be improved through an updated & rationalized communication strategy to address refusal and vaccine hesitancy by adopting an innovative approach for evidence-driven messaging. The visible involvement of the elected representatives, religious intellectuals, societal influencers, teachers, showbiz & sports celebrities, and healthcare officials must continue to be an essential approach to maintain public confidence and promote the HPV vaccine as a routine preventative measure in the country. Expanding fixed EPI sites for HPV vaccination, promoting school-centered vaccination, rationalizing outreach in marginalized areas, sustaining the cold chain system, implementing a culturally acceptable communication plan, and resolving internet connectivity challenges are the key strategies to address implementation challenges.

The long-term sustainability of this initiative depends on steady domestic funding, close collaboration between the Ministries of Education and Health, and the integration of the HPV vaccine into Pakistan’s routine immunization schedule. Increasing HPV vaccine uptake through campaigns and routine immunization can be significantly improved by addressing these implementation gaps, bringing the country closer to eradicating cervical cancer as a major global and national public health issue.

### Data Limitations

The findings are based on administrative data generated during the HPV vaccination campaign as recorded in the EPI-MIS system, which may be subject to over- or under-reporting and were analyzed without adjustment or extrapolation.

## Figures and Tables

**Table 1:** Details of all the HPV-related cancer cases reported in Pakistan (2021–2023).

Province/ Federally Administered Area	Cancer Registry/Hospital/Institution Name	2021	2022	2023	Total Cases
Cervical Cancer					
Punjab	Punjab Cancer Registry (Shaukat Khanum Memorial Cancer Hospital & Research Center), Lahore	179	113	114	406
	2. Faisalabad Medical University Hospital, Faisalabad	35	50	41	126
	3. Nishtar Medical University Hospital, Multan	4	24	39	67
	4. Multan Institute of Nuclear Medicine and Radiotherapy (MINAR) Cancer Hospital, Multan	109	110	83	302
	5. Sheikh Zayed Hospital, Rahim Yar Khan	18	24	24	66
	6. DG Khan Medical College affiliated with Allama Iqbal Teaching Hospital, DG Khan	12	12	12	36
	7. Quaid-e-Azam Medical College affiliated Hospital, Bahawalpur	3	4	3	10
	8. Quaid-e-Azam International Hospital, Rawalpindi	0	0	0	0
	Total Cases	360	337	316	1013
Sindh	9. Karachi Cancer Registry (Aga Khan University Hospital, Karachi)	52	54	35	141
	10. Dow University Hospital, Karachi	6	12	3	21
	11. Neurospinal & Cancer Care Institute (M. Hashim Memorial Trust) NCCI, Karachi	4	4	7	15
	Total Cases	62	70	45	177
Balochistan	12. Center for Nuclear Medicine and Radiotherapy (CENAR), Quetta	18	21	09	48
	Total Cases	18	21	09	48
Islamabad	13. Pakistan Institute of Medical Sciences, Islamabad (PIMS)	6	11	11	28
	14. PAF Hospital, Islamabad	1	1	1	3
	15. Shifa International Hospital, Islamabad	27	12	18	57
	Total Cases	34	24	30	88
Azad Jammu & Kashmir (AJK)	16. AJK Medical College affiliated Hospitals, Muzaffarabad	0	0	2	2
	17. Combined Military Hospital, Muzaffarabad	0	0	2	2
	Total Cases	0	0	4	4
	Grand Total (Year Wise)	473	452	404	1330
Khyber Pakhtunkhwa (KPK), Azad Jammu & Kashmir (AJK), Gilgit Baltistan (GB)	18. North West Cancer Registry (Shaukat Khanum Memorial Cancer Hospital & Research Center, Lahore)	250			250
	Grand Total (All Cases)				1580
Other HPV-related Cancer					
Punjab	Sheikh Zayed Hospital, Rahim Yar Khan	25	30	26	81
	Total Cases	25	30	26	81
Balochistan	2. Center for Nuclear Medicine and Radiotherapy (CENAR), Quetta	15	07	15	37
	Total Cases	15	07	15	37
	Grand Total	40	37	41	118

**Table 2:** HPV Vaccination Campaign Progress Analysis (Day 1–12 and Mop-Up Phase).

Province	9 Years Girls			10–14 Years Age Girls			Total 9–14 Years Girls		
	Target	Number of Girls Vaccinated	Coverage (%)	Target	Number of Girls Vaccinated	Coverage (%)	Target	Vaccinated	Coverage (%)
Punjab	1,318,943	1,290,538	98%	7,217,211	5,587,773	77%	8,536,154	6,878,311	81%
Sindh	630,398	537,371	85%	3,449,513	2,137,156	62%	4,079,911	2,674,527	66%
AJK	42,140	21,165	50%	230,585	95,487	41%	272,725	116,652	43%
Islamabad	22,696	11,103	49%	124,192	44,910	36%	146,888	56,013	38%
National	2,014,176	1,860,177	92%	11,021,502	7,865,326	71%	13,035,678	9,725,503	75%

**Table 3:** District-wise Coverage During HPV Campaign (15–27 September) & Catch-Up Days.

Province	Total Districts	<60%	60–69%	70–79%	≥80%
Punjab	36	0	0	7	29
Sindh	30	7	2	4	17
AJK	10	9	1	0	0
Islamabad	2	2	0	0	0
Total	78	18	3	11	46

**Table 4:** Girls Missed During HPV Campaign (15–27 September) Days &, Targeted in Catch-Up Days.

Missed Girls Information (Cumulative)						
Province	Not Available (NA)	Refusals (R)	Sick (S)	Total Missed Girls	Around Missed Girls Vaccinated	(%)
Punjab	648,247	2,731,158	23,171	3,402,576	1,016,937	29.89%
Sindh	216,834	1,061,160	24,609	1,302,603	115,897	8.90%
AJK	9477	156,910	586	166,973	9972	5.97%
Islamabad	6556	69,336	273	76,165	4102	5.39%
Total	881,114	4,018,564	48,639	4,948,317	1,146,908	23.18%

**Table 5:** Girls Reached (%) by HPV Vaccination Strategy.

Province	Fixed		Schools		Community	
	No	%	No	%	No	%
Punjab	224,003	3.50%	2,346,708	36.80%	3,811,581	59.70%
Sindh	175,593	6.60%	333,712	12.50%	2,162,847	80.90%
AJK	0	0%	55,119	56.60%	42,276	43.40%
Islamabad	583	1.20%	22,028	44.80%	26,518	54%
Total	400,179	4.30%	2,757,567	30%	6,043,222	65.70%

**Table 6:** Rapid Convenience Assessment (RCA) of Immunization Coverage during Campaign (15–27 September) & Catch-Up Days.

Province	Expected RCA	Conducted RCA	Communities Identified for Mop-Up
Punjab	62,400	37,200 (60%)	15,514 (42%)
Sindh	25,110	13,362 (53%)	2941 (22%)
AJK	6855	533 (8%)	222 (42%)
Islamabad	1080	355 (33%)	270 (76%)
Total	95,445	51,450 (54%)	18,947 (37%)

**Table 7:** Sources Of Campaign Information & Reasons for Non-Vaccination.

Sources of Campaign Information		Reasons for Non-Vaccination	
Variable	Percentage	Variable	Percentage
Health Worker	31%	Refusal	71%
Mosque Miking	21%	Not Available	22%
Mobile Miking	18%	Sick	3%
Fellow School Children	8%	Other Reasons	2%
Posters	9%	Parent Unaware	1%
Television	7%	No Campaign Information	1%
Newspaper	4%		
Other Sources	2%		

**Table 8:** AEFI Cases Reported by Province During Phase I of the HPV Vaccine Introduction.

AEFI Cases	Punjab	Sindh	AJK	Islamabad	Total
Reported cases	29	102	44	15	190
Mild	29	72	44	15	160
Serious *	-	30	-	-	30

* *Investigated*.

## Data Availability

The data will be available from the corresponding author upon reasonable request.
